# Unmet Medical Needs in the Management of Ulcerative Colitis: Results of an Italian Delphi Consensus

**DOI:** 10.1155/2019/3108025

**Published:** 2019-09-02

**Authors:** Marco Daperno, Alessandro Armuzzi, Silvio Danese, Walter Fries, Giuseppina Liguori, Ambrogio Orlando, Claudio Papi, Mariabeatrice Principi, Fernando Rizzello, Angelo Viscido, Paolo Gionchetti

**Affiliations:** ^1^Gastroenterology Unit, Mauriziano Hospital, Turin, Italy; ^2^IBD Unit, Fondazione Policlinico Universitario A. Gemelli IRCCS, Rome, Italy; ^3^Università Cattolica del Sacro Cuore, Rome, Italy; ^4^Humanitas Research Hospital, Milan, Italy; ^5^Clinical Unit for Chronic Bowel Disorders, Department of Clinical and Experimental Medicine, University of Messina, Messina, Italy; ^6^I&I Medical Advisor, Pfizer srl, Rome, Italy; ^7^IBD Unit, Division of Internal Medicine, “Villa Sofia-Cervello” Hospital, Palermo, Italy; ^8^IBD Unit, S. Filippo Neri Hospital, Rome, Italy; ^9^Gastroenterology Section, Department of Emergency and Organ Transplantation, University of Bari Aldo Moro, Bari 70124, Italy; ^10^IBD Unit, Department of Medicine and Surgery (DIMEC), University of Bologna, S. Orsola-Malpighi Hospital, Bologna, Italy; ^11^Department of Life, Health, and Environmental Sciences, University of L'Aquila, L'Aquila, Italy

## Abstract

**Background:**

The lifelong and remitting nature of ulcerative colitis results in considerable disability and a substantial negative impact on quality of life. The major goal of the therapy of ulcerative colitis is considered to be the modification of the course of the disease, so that the patient's quality of life can be improved while minimising disease-related disability. Although considerable progress in understanding the molecular pathways involved in ulcerative colitis has led to improved treatment options, there is currently no definitive cure for ulcerative colitis, there remain considerable unmet needs in terms of long-term efficacy and safety, and there are many patients who continue to be burdened by physical and psychological symptoms. Defining unmet needs can help to increase the awareness of the shortcomings of current therapeutic management and highlight the need to achieve not only a control of clinical symptoms but also control of mucosal healing, in order to attain the best possible long-term outcomes.

**Methods:**

With the aim of providing a better understanding of the unmet needs of patients towards improving overall care, a Delphi process was used to obtain consensus among a group of Italian ulcerative colitis experts. The consensus group met with a major focus of delineating the unmet needs of current treatment strategies and overall management of ulcerative colitis, while also focusing on quality of life and patient care.

**Results:**

Three main areas were identified: (i) treatment, (ii) monitoring and risk management, and (iii) patient-related issues. A high level of consensus was reached on all but one of the statements identified.

**Conclusions:**

The findings arising from the Delphi process provide valuable insights into the unmet needs in the management of moderate-to-severe ulcerative colitis from the clinician's perspective, while emphasising the benefits of therapeutic individualization and suggesting areas that need additional study with the aim of optimising the treatment of patients with ulcerative colitis.

## 1. Introduction

Ulcerative colitis (UC) is a chronic inflammatory disease of the bowel with a course that is lifelong and remitting [1]. As such, the disease results in significant disability with a substantial negative impact on the overall quality of life of sufferers. While its precise aetiology remains unknown, it is likely to be multifactorial encompassing a range of aspects that include genetic predisposition, deficits in the epithelial barrier, and abnormal dysregulation of immune responses, along with environmental factors [1]. At present, there is no definitive cure for UC, although in recent decades exceptional progress has been made in better understanding the molecular pathways involved. In this regard, the known involvement of multiple inflammatory pathways has allowed for new therapeutic advances and novel treatments for UC [2]. In particular, the availability of biologics had given clinicians the opportunity to optimise the overall management of the disease, and patients are now undergoing lower rates of surgery and have better clinical and patient-reported outcomes in the long term compared to the past [2].

With these advances in mind, the major goal of the therapy of UC is now considered to be the modification of the course of the disease, so that the patient's quality of life can be improved while minimising disease-related disability [3, 4]. Clinicians must also balance the benefit/risk ratio when selecting treatments [3]. In order to better disease outcomes, a treat-to-target approach is currently advocated [4]. This can be considered as a novel strategy that is already used not only in other inflammatory disease but in other chronic pathologies that require lifelong management [4]. In such an approach, clinicians use actively sought objective measures of disease activity that are then used to direct specific and successive measures to keep the disease under control [4]. In UC, measures of disease activity include not only the presence of macroscopic lesions but also patient-reported symptoms and signs. Indeed, the most recent European guidelines on the management of UC fully recommend the routine use of a treat-to-target strategy for UC [5]. The revised guidelines advocate that the goal of treatment is to obtain resolution of both symptoms and signs of inflammation [5].

As mentioned, the currently available therapeutic armamentarium has now begun to dramatically revolutionise the management of UC, reversing the lifelong outlook of the disease [6]. Notwithstanding the large number of therapeutic options available, there are still concerns regarding unmet needs in terms of long-term efficacy and safety, which have the potential to greatly impact the overall quality of care and, consequently, the patient's quality of life [5]. Among the major concerns, for example, around one-third of UC patients do not have an adequate response to treatment or will lose response to therapy at a rate of 10-20% per year [6]. The current treatment options have been expanded with the availability of JAK inhibitors, small molecules that inhibit the signal transduction of several cytokines to reduce the inflammatory response [6].

There are also many patient-associated features and unmet needs that clinicians must consider. Unfortunately, many patients with moderate-to-severe UC treated with conventional therapies report that their condition is not well-controlled and about one-fourth report that the disease seriously impairs quality of life and work productivity [7]. In fact, patients continue to experience a sizeable number of symptoms and are thus burdened by both physical and psychological symptoms, which are even more pronounced during the active phase of the disease [8]. This highlights the need to broaden the evaluation of patients so that it encompasses multiple symptoms, including those related to fatigue and psychological aspects [8, 9]. Quality of life issues are crucial to the management of UC patients, and higher disease activity has been related with poorer health-related quality of life and impairment of work and daily activities, as well as increased healthcare costs [10, 11].

Considering the unmet needs in UC, a number of studies have been performed to better define them [7, 11–13]. This is a highly relevant issue for practice, as several factors could potentially limit the optimal management of patients with UC, and a better understanding of the unmet needs in patient management can help to improve overall care [12]. Such considerations are of particular importance when evaluating a patient's acceptance of their disease and its treatment [12].

All these concerns pose reasonable challenges to healthcare providers, for which at present there are no definite answers. In order to identify and clearly define the unmet needs in the overall management of moderate-to-severe UC, a Delphi process was used to obtain consensus among a group of Italian experts. The major focus of the consensus group was to delineate the unmet needs of current treatment strategies and overall management, while focusing on quality of life and patient care. Three main areas were identified: (i) treatment, (ii) monitoring and risk management, and (iii) patient-related issues.

## 2. Materials and Methods

### 2.1. Motivations for Choice of Delphi Methodology

Given the diversity of opinion that can occur with the diagnosis and management of disease, formal group consensus methods are able to organise both objective and subjective judgments. Accordingly, such methods can provide guidance when there is limited evidence [14]. Formal group consensus methods allow for the inclusion of a wide range of knowledge and experience, permitting interaction between members while stimulating constructive debate and preventing influential behaviour of opinion to formulate suggestions about specific questions of interest [14]. Since perfect agreement is seldom reached, the Delphi consensus methodology has the main objective of identifying a central propensity in the group and providing the level of agreement reached [14]. Considering these aspects, the Delphi process was chosen as the current scenario in the management of UC closely matches the overall objectives of the current project.

The Delphi method attempts to do this using a series of well-defined questionnaires that are modified based on successive rounds of voting and feedback [15]. Responses are collated by the organisers and sent back to participants in summary form, usually indicating the judgement of the group along with the initial judgement. Participants can then revise their judgement in light of the group feedback. This process may be repeated a number of times and is usually conducted over 2 to 4 “rounds” with the results elicited, tabulated, and reported to the group after each round. The Delphi consensus method is now widely used in the development of review-based consensus methodology [16].

### 2.2. Steering Committee

The steering committee met via web in March 2018 to discuss pertinent issues in the current management of UC. Members of the steering committee were identified based on their interest on the topic, their overall level of knowledge and clinical experience, and their motivation to share their knowledge and experience using the Delphi method.

### 2.3. Voting Platform

An Internet-based web platform was established in which voting was carried out for each statement. Access to the platform was allowed via username and password, which were provided to each member. Members could vote on each statement (on a 1 to 9 scale: 1-3 meaning a disagreement; 4-6, neither agreement nor disagreement; 7-9, agreement), and a comment box was available in which feedback could be provided.

### 2.4. Phase 1

In April 2018, members of the steering committee were asked to produce statements on the unmet needs of UC and accompanied by essential supporting literature. Similar ideas were clustered together, and the statements were reviewed independently and refined by a Health Research Methodology expert, taking into consideration the overall consensus and comments for each statement. The Health Research Methodology expert also carried out a literature search in PubMed to search for additional supporting literature for each statement; 594 records were retrieved spanning the period from January 1, 2013 to May 30, 2018. Of the 22 statements received, 7 duplicates were removed for a total of 15.

### 2.5. Phases 2 and 3

In Phase 2 in June 2018, the steering committee voted via web platform on the 15 statements from Phase 1. Voting was carried out in the same manner, and an independent Health Research Methodology expert reviewed the overall consensus and refined the statements accordingly, to obtain 13 statements. These statements were voted on again by the steering committee members in August 2018 (Phase 3), and after review by an independent Health Research Methodology expert, a total of 11 statements were obtained.

### 2.6. Delphi Process

The panel of 68 potential specialists for voting was recruited among experts in the field of UC, chosen by two independent experts in Health Research Methodology based on clinical experience and scientific production. Of the 68 experts identified and contacted via email, 41 (60%) replied and agreed to be involved to the Delphi voting consensus process, which was opened in October 2018 and lasted one month. Credentials to access the platform were emailed to participants. Voting on the platform was carried out via a dedicated web platform, again on a 1 to 9 scale. Consensus was considered when ≥75% of the voting specialists agreed or disagreed with the statement. All 41 participants who agreed to give their opinion accessed the platform and voted on all 11 statements.

## 3. Results

The composition of the steering committee and selection of the voting panel has been described in Materials and Methods. The steering committee drafted initial statements regarding unmet needs that were then revised independently. The statements covered three major areas: treatment, monitoring and risk management, and patient-related issues. The initial statements were drafted by the steering committee, and a total of 15 statements were agreed upon and subjected to the first round of voting via the web. Statements were refined based on feedback provided by the committee members. Some statements were merged as they were deemed to be somewhat redundant, and 13 statements were then subjected to a second round of voting by the steering committee. These statements were modified by the steering committee based on feedback and were reduced to 11 statements. Finally, the 11 statements were voted on by a panel of 41 experts who agreed to be involved in the current Delphi process. The final statements and voting results are shown in Table 1.

Broad consensus (≥75%) was reached for all statements except for statement #9 on the need for effective and appropriate strategies that can limit the risk of developing colorectal cancer. Over 95% of the participants believe that there is a need for the following: (i) a treatment strategy that can induce sustained corticosteroid-free remission and mucosal healing in the majority of patients; (ii) drugs that are associated with only minimal or no loss of response; and (iii) individualised treatment based on reliable predictors of response.

## 4. Discussion

The main goal of the present Delphi consensus was to provide clear definitions for the current unmet needs in the overall management of UC, with a focus on moderate-to-severe disease. The process used reached a high level of agreement for all except one of the statements subjected to voting by the panel of 41 experts, indicating that the process used was effective in obtaining consensus on a wide range of topics related to treatment strategies, monitoring and risk management, and patient-related issues in the management of moderate-to-severe UC. In addition, the current Delphi attempted to focus on patient-related aspects and quality of care. The results of voting for each of the statements in the three areas identified are discussed below.

### 4.1. Treatment

Consensus for statement 1 reached a very high level of consensus (95.1%). This indicates that the experts felt that it is important that a treatment strategy for UC induce sustained corticosteroid-free remission and mucosal healing, at least in the majority of patients. In agreement with this concept, according to European Crohn's and Colitis Organisation (ECCO) guidelines, the goal of maintenance therapy in UC is to maintain complete and stable steroid-free remission [5]. In fact, in UC, mucosal healing has been associated with fewer major surgeries and fewer hospitalisations [17]. Notwithstanding, newer endoscopic techniques have highlighted the possibility that even among patients with normalised endoscopic features, a proportion of tissue with residual microscopic activity is relevant for subsequent relapses [18–21]. Furthermore, long-lasting action is an aspect that is considered by patients to be very important, and it is one of the most frequently cited items when considering patient preferences for treatment [3, 4]. Unfortunately, at present, in real-world scenarios, most patients with UC do not achieve composite clinical and endoscopic remission [3]. Thus, considering the above, a treatment strategy that can induce sustained clinical and endoscopic remission is clearly desirable [22].

Just over 75% consensus was reached for statement 2 regarding the need for a therapy with a rapid onset of action. Recent studies have shown that a rapid onset of action and effectiveness in inducing mucosal healing are important advantages of treatment [5]. Evidence to support this statement also comes from real-world studies, where a rapid onset of action is among the attributes most frequently considered to be important by UC patients [11, 23]. Remission should thus be as quick as possible to improve the patient's quality of life. The time to achieve remission will vary among different therapeutic approaches. Steroids tend to have a rapid clinical effect with remission seen within two weeks in many patients [24]. Antitumour necrosis factor- (TNF-) *α* agents have become a significant advance in the management of UC, as they are associated with rapid clinical and endoscopic remission [25]. Among newer agents, the JAK inhibitor tofacitinib is worthy of mention as it has been observed that it has a rapid onset of action, reducing symptoms of UC within 3 days [26], while the monoclonal antibody vedolizumab has a relatively slow onset of action [27]. As highlighted in recent guidelines, rapid and sustained onset of action may help to reduce the potential for the development of chronicity [5], but the reason for the somewhat weak agreement lies within the clinical observation that being a lifelong disease, in addition to the absolute short time in inducing remission, which is obviously desirable, sustainability of remission in UC is more desirable as underlined in the following statements.

A high level of consensus (95.1%) was also reached for statement 3 on the need for drugs that are associated with only minimal or no loss of response. Treatment recommendations for UC consider that the target for UC should comprise both clinical/patient-reported outcome remission and endoscopic remission, with histological remission as an adjunctive goal [4]. However, real-world studies have shown that most patients with UC do not achieve such a composite endpoint [3]. While current medical therapies, including biologicals, have established efficacy in inducing and maintaining remission, they also have several limitations such as lack of primary response and loss of response during the maintenance phase, in addition to safety concerns for some drugs [28]. For example, many patients treated with biologics experience loss of response due to antidrug antibodies within the first 12 months of therapy, and a concomitant immunomodulator to reduce the immunogenicity is required [29]. At present, none of the currently available drugs actually have a curative role or address the underlying pathophysiology [28].

The experts further agreed that there is a need for therapies that can effectively treat moderate-to-severe disease (statement 4, 80.5% consensus). The incidence of UC varies depending on the geographic region with a reported incidence that ranges from 1.2 to 20.3 cases per 100,000 persons per year, and is considered common in both North America and Western Europe [30]. Moderate-to-severe UC is associated with increased morbidity, as it has a major impact on health-related quality of life and impairment of both social and professional activities [7]. Current treatment options for UC are evolving, and the most appropriate treatment will depend on the severity of disease and patient preferences. Despite a large number of medical treatment options available, changes to therapy are frequent in patients with moderate-to-severe UC, and relapse rates are considered to be high [31]. Moreover, approximately 15% of patients will still require proctocolectomy [30]. Many patients with moderate-to-severe UC who are treated with conventional therapies continue to report that their disease is not controlled and one-fourth report unmet clinical needs, with grave impairment of both quality of life and employment [32]. Accordingly, achieving remission is associated with improved quality of life, less disability, and better work-related outcomes [32]. When considering the effectiveness of therapy, the patient's assessment of efficacy may influence their adherence to treatment [33]. Nonadherence to therapy is common in UC, and is associated with increased rates of relapse and disability [34, 35]. In this regard, simplified therapeutic regimens have the potential to improve adherence and associated outcomes [35]. Given all of the above, the experts agreed that there remains a need for therapies that can effectively treat moderate-to-severe UC.

The experts agreed that there is an unmet need for individualised treatment based on reliable predictors of response (statement 5, 95.1% consensus). Anti-TNF-*α* agents and vedolizumab are used in cases of moderate-to-severe UC refractory to conventional treatment with corticosteroids and/or immunosuppressants [5], and primary and secondary nonresponses, as mentioned, are rather heterogeneous [36]. Clinical phenotypes are known to vary in patients with UC, and these different phenotypes will often entail the use of different strategies for management [37]. A number of molecular markers have been evaluated in terms of their ability to predict responsiveness to biologics, including genetic, serological, histological, and faecal markers [37]. However, while some of these markers have suitable sensitivity and specificity, more studies are needed to identify more robust markers and apply them in routine clinical practice, with the aim of achieving a more precision-based approach.

More accurate diagnostic tools also have the potential to help select patients for the most appropriate therapy based on specific pathways, and identification of factors that can help predict response to anti-TNF-*α* drugs in UC would also help to minimise both risk and costs for those who are not predicted to respond. The possibility of using therapeutic drug monitoring to assess one or more of these parameters has been broadly suggested, although at present the data would appear to be insufficient to propose a standardised algorithm [38]. To date, a multitude of factors related to the patient, disease, and treatment have been investigated, although at present there is very limited use of predictive factors in routine clinical practice [39, 40].

The experts further found consensus for statement 6 on the need for a therapeutic strategy that can reduce hospitalisation and need for surgery (85.4% consensus). In fact, UC is a chronic inflammatory disease with severe consequences. These include the need for hospitalisation and colectomy, which have significant burden on both quality of life and costs of management [7, 41]. Mucosal healing is now considered as a crucial endpoint in the management of UC, and, moreover, is a strong predictor of fewer surgeries and a reduced number of hospitalisations [22]. This suggests that mucosal healing might be considered as a preferred clinical endpoint in patients with moderate-to-severe UC. The effectiveness of such treatments was shown in the population-based study by Burisch et al. [42]. Patients who were treated more aggressively with immunomodulators and biological therapy had similar outcomes to historical cohorts from the past two decades; notwithstanding, therapy with immunomodulators was associated with lower rates of hospitalisation [42]. Fewer hospitalisations are also undoubtedly associated with substantial savings in healthcare costs and improved quality of life.

### 4.2. Monitoring and Risk Management

Considering the area of monitoring and risk management, consensus was reached regarding the need for validated, noninvasive methods to monitor disease activity (statement 7, 75.6% consensus). It is evident that UC has a relapsing and remitting course; furthermore, inflammatory status has utility in the evaluation of disease activity and in individualisation of treatment [43]. Unfortunately, at present no simple diagnostic assay is available that can routinely be used to monitor gastrointestinal inflammation, even if noninvasive markers can provide an indirect status of disease activity [43]. While histopathological and endoscopic examinations can faithfully evaluate inflammatory status, these techniques are not suited for routine applications as they are invasive, time-consuming, and costly. Imaging techniques such as ultrasound and Doppler have been assessed, but they are of little utility in UC [43]. Thus, in the absence of better tools to assess disease activity, clinical activity indices are widely used. In the attempt to define a noninvasive method, several markers have been studied, including calprotectin, lactoferrin, radiolabelled leukocytes, calgranulin C, and pyruvate kinase type M2 [39, 41]. Faecal calprotectin has also been investigated and may be one of the most promising noninvasive markers for monitoring disease activity in UC to date [44]. It is clear that the ideal noninvasive marker would need to be highly accurate, easy to assay, and cost-effective.

Statement 8 held that there is an unmet need for management strategies that have a better benefit/risk ratio (75.6% consensus). Indeed, a wide range of pharmacological agents is used to manage UC, and given their diversity are associated with a broad range of adverse effects and safety concerns. In this regard, guidance for their use with a focus on safety has been developed based on current knowledge and clinical experience [45]. Conventional agents are associated with a number of safety concerns with the possibility of the involvement of multiple organ systems [45], and both conventional and biological agents may carry an additional concern of malignancy [45, 46]. In general, all biological agents (infliximab, adalimumab, golimumab, and vedolizumab) have been considered as relatively safe, and also tofacitinib, a JAK inhibitor recently approved for UC, appears to have a safety profile similar to that reported for patients with UC treated with biologic agents, except for a possibly higher incidence of dose-dependent herpes zoster infection [47]. Nevertheless, adequate screening and precautions are needed before their initiation, and regular surveillance is strongly recommended to minimise the risk of infections and other adverse events in UC patients [46].

The only statement for which consensus was not reached was statement 9 on the need for effective and appropriate strategies that can limit the risk of developing colorectal cancer (61.0%). It is known that there is an association between UC and the development of colorectal cancer [48]. The risk for colorectal cancer begins to significantly increase at 8-10 years after diagnosis of UC; the risks for developing colorectal cancer are linked with younger age at diagnosis, longer duration of disease, the anatomical extent of intestinal involvement, inflammatory status, and family history for colorectal cancer [48]. Surveillance programs are recommended in most settings to detect the disease at an early stage and reduce the risk of mortality [48]. While colonoscopy with random biopsy has been used traditionally [49], more recent evidence has suggested that chromoendoscopy may be a better tool to detect neoplasia [50]. Improved knowledge of precancer in UC along with technological advances has also led researchers to consider the possibility to use molecular markers for detection in screening and prevention [51]. Reduction of inflammation using 5-aminosalicylic acid agents reduces the risk of developing colorectal neoplasia; when administered with additional strategies to control inflammation, these agents are held to be of benefit and are recommended [49, 52]. At present, there is insufficient evidence to support a chemoprotective effect of purine analogues or anti-TNF-*α* medications [49, 52]. However, consensus was not reached regarding the need for more effective and appropriate strategies for the prevention of colorectal cancer, perhaps because this cannot be considered as a priority at present. This may be due to a number of factors, such as greater awareness that chronic inflammation is associated with an increased risk of colorectal cancer, increased physician confidence that achieving mucosal healing reduces the risk of colorectal cancer, that the specific risk of disease is believed to be lower than previously suspected [53], and that some of the therapies utilized likely have a chemopreventive effect on colorectal cancer [49, 52].

### 4.3. Patient-Related Issues

Patient preferences for therapies have an impact on virtually every aspect of healthcare. In line with this philosophy, the experts agreed with statement 10 that there is a need for therapies which are more compatible with patients' expectations and comorbidities (80.5% consensus). The clinical effectiveness of treatments in terms of symptom control and risk of flare-up is one of the most preferred aspects as it has a major impact on the quality of life [33]. Moreover, there is increasing awareness that patient preferences for treatment, considering both efficacy and adverse effects, play an important role when establishing a therapeutic plan [32]. Indeed, it is now generally agreed that patient preferences for treatment can improve adherence to therapy and treatment outcomes in UC [33]. Not unsurprisingly, therapies that are effective, long lasting, and with a rapid onset of action are highly preferred by patients as they are more effective in helping them meet their expectations for treatment [11, 32, 33]. The control of symptoms is important since patients with inflammatory bowel disease as UC may also present with anxiety and depression. These symptoms are common and are linked to disease-related disability [11, 32, 33, 47, 54]. In addition, treatments that are better tolerated will have fewer effects on existing comorbidities. Patients with UC are now faced with an increasing number of choices regarding the management of the chronic illness, and patient-stated preference is an increasingly used methodology that can help to establish the relative utility for different therapeutic options based on efficacy, adverse effects, and other treatment attributes such as route and frequency of administration [32]. As a result, patient preferences carry growing importance when making decisions regarding their care.

The last statement on patient-related issues concerned the agreed need for consensus on the assessment of quality of life, fatigue, psychological symptoms, social problems, and disability (85.4%). These aspects are now receiving greater attention. Patients with UC are burdened by impaired physical health and impaired mental health, as well as pain, problems with anxiety, social and physical functioning, depression, and sleep disturbance [7, 9, 32, 55–57]. Poor sleep quality is further related to depression, and fatigue is considered to be one of the bothersome symptoms of inflammatory bowel disease [7, 9, 32, 55–57]. Thus, patient assessment must be comprehensive and multidimensional. While the wide range of patient-related burden is becoming increasingly clear, there is still little consensus regarding its routine assessment in daily practice, and the most recent ECCO recommendations provide no guidance in this regard [5]. Assessment of quality of life is an important aspect of all management choices, with the improvement of the quality of life as a key goal of therapy. There are multiple domains of disease activity assessment in UC, and targets within each domain need to be recognised with clearly established goals [58].

## 5. Conclusions

The value of the present Delphi consensus statements lies in the more precise delineation of the unmet needs in the management of moderate-to-severe UC from the clinician's perspective, while emphasising the benefits of therapeutic individualisation. By defining the unmet needs in the treatment of UC, this can help to increase awareness of the shortcomings of therapeutic management, and highlight the need to achieve, whenever possible, not only control of clinical symptoms but also mucosal healing, in order to attain the best possible long-term outcomes. Quality of life should also be given primary consideration when planning treatment. Lastly, the consensus statements aid in further defining areas that need additional future study and which warrant further consideration by clinicians in the treatment of UC.

## Figures and Tables

**Table 1:** Final statements and voting results.

		% consensus
*Treatment*		
1	There is a need for a treatment strategy that can induce sustained corticosteroid-free remission and mucosal healing in the majority of patients	95.1
2	There is a need for a therapy with rapid onset of action	75.6
3	There is a need for drugs that are associated with only minimal or no loss of response	95.1
4	There is a need for therapies that can effectively treat moderate-to-severe disease	80.5
5	There is an unmet need for individualised treatment based on reliable predictors of response	95.1
6	There is an unmet need for a therapeutic strategy that can reduce hospitalisation and need for surgery	85.4

*Monitoring and risk management*		
7	There is an unmet need for validated, noninvasive methods to monitor disease activity	75.6
8	There is an unmet need for management strategies with better benefit/risk ratio	75.6
9	There is a need for effective and appropriate strategies that can limit the risk of developing colorectal cancer	61.0

*Patient-related issues*		
10	There is an unmet need for therapies that are more compatible with patients' expectations and comorbidities	80.5
11	There is a need for consensus regarding assessment of quality of life, fatigue, psychological symptoms, social problems, and disability	85.4

## Data Availability

The general dataset is available on request by writing to the corresponding author, Alessandro Armuzzi.
